# Iodine and Aconite in Periodontitis

**Published:** 1869-06

**Authors:** 


Iodine and Aconite in Periodontitis.—Professor Abbott writes: The best remedy, and the one that works the most conveniently for periodontitis, I have ever used, is a mixture of equal parts of officinal tincture of iodine and tincture of aconite root applied to the gum around the roots of the tooth with a camel's hair brush, or a portion of cotton-wool at the end of a stick. I have been using it for a year, and have not found it fail. I apply it, in the early stages of the inflammation, once in twenty-four hours, and in very severe cases twice.—*Boston Journal*, January 7.—(*Quœre:* What dose of aconite is administered?)

